# Activation of oxidoreductases by the formation of enzyme assembly

**DOI:** 10.1038/s41598-023-41789-9

**Published:** 2023-09-01

**Authors:** Tomoto Ura, Nanako Sakakibara, Yu Hirano, Taro Tamada, Yoichi Takakusagi, Kentaro Shiraki, Tsutomu Mikawa

**Affiliations:** 1 Institute for Quantum Life Science, National Institutes for Quantum Science and Technology, 4-9-1 Anagawa, Inage-ku, Chiba, 263-8555 Japan; 2 Institute of Pure and Applied Sciences, University of Tsukuba, 1-1-1 Tennodai, Tsukuba, Ibaraki 305-8573 Japan; 3 RIKEN Center for Biosystems Dynamics Research, 1-7-22 Suehiro-cho, Tsurumi-ku, Yokohama, Kanagawa 230-0045 Japan; 4 Department of Quantum Life Science, Graduate School of Science, Chiba University, Yayoi-cho, Inage-ku, Chiba, 263-8522 Japan

**Keywords:** Enzymes, Proteins

## Abstract

Biological properties of protein molecules depend on their interaction with other molecules, and enzymes are no exception. Enzyme activities are controlled by their interaction with other molecules in living cells. Enzyme activation and their catalytic properties in the presence of different types of polymers have been studied in vitro, although these studies are restricted to only a few enzymes. In this study, we show that addition of poly-l-lysine (PLL) can increase the enzymatic activity of multiple oxidoreductases through formation of enzyme assemblies. Oxidoreductases with an overall negative charge, such as l-lactate oxidase, d-lactate dehydrogenase, pyruvate oxidase, and acetaldehyde dehydrogenase, each formed assemblies with the positively charged PLL via electrostatic interactions. The enzyme activities of these oxidoreductases in the enzyme assemblies were several-folds higher than those of the enzyme in their natural dispersed state. In the presence of PLL, the turnover number (*k*_cat_) improved for all enzymes, whereas the decrease in Michaelis constant (*K*_M_) was enzyme dependent. This type of enzyme function regulation through the formation of assemblies via simple addition of polymers has potential for diverse applications, including various industrial and research purposes.

## Introduction

Enzymes have been studied not only as intracellular catalysts but also because of their wide range of industrial applications^1,2^. Enzymes’ versatility stems from their high substrate specificity, and ability to catalyze reactions efficiently under relatively mild conditions of temperature and pressure. There are a few enzymes whose activities differ greatly between in vivo and in vitro conditions^3–5^, and in some cases they do not exhibit the expected activity under conditions of practical applications. Therefore, several methods, such as protein engineering^6,7^ and chemical modifications^8,9^, have been proposed to modify enzymes to adapt to the extracellular environment. These are powerful methods for creating altered versions of the wild-type enzymes that are tailored to adapt to the environment of extracellular applications. However, these methods also have certain drawbacks; random mutagenesis and enzyme screening are time-consuming and costly. Therefore, basic research that leads to the establishment of a simple method of enhancing enzyme activity is necessary.

Activities of many enzymes are known to be regulated by interaction with highly charged macromolecules such as RNA^10,11^ and intrinsically disordered regions (IDRs) of proteins^12,13^. Based on these reports, we investigated enzyme activity modulation through enzyme-polymer interaction in vitro, which may pave the way for an alternative technology for enzyme activation. We have previously shown that the negatively charged l-lactate oxidase (LOX, p*I* = 6) can form enzyme assemblies in the presence of the positively charged poly-l-lysine (PLL), a polyelectrolyte that mimics IDRs at physiological pH, and trigger the activation of LOX^14^. Enzyme assemblies have been investigated as a mechanism of intracellular enzymatic activation^15,16^. The size of an enzyme assembly is important for enzyme activation^14^. Assembly sizes ranging from several tens to hundreds of nanometers had a higher activation effect than those on the scale of several micrometers. With the existing data of enzymological analyses, clarifying the effect of enzyme assembly formation on enzymatic activities is difficult because there are no established methods for detecting the structure and the state of the enzyme active sites in the enzyme assemblies. It is also important to determine whether similar electrostatic interaction-based enzyme assemblies and activation occur in other enzymes.

In this study, we examined the effect of formation of enzyme assemblies on the activities of five oxidoreductases and experimentally demonstrated that their activities were elevated to a great extent by the formation of enzyme assemblies. An increase in turnover number (*k*_cat_) was common to all tested enzymes, and an increase or decrease in the Michaelis constant (*K*_M_) was dependent on the physicochemical properties of the enzyme. It is notable that the addition of polyelectrolytes to the enzyme solution can enhance its activity and that this technique is less laborious than other conventional methods and does not require time-consuming screening of mutants.

## Results

### Activation of oxidoreductases by electrostatic interaction with PLL

**Figure 1:**
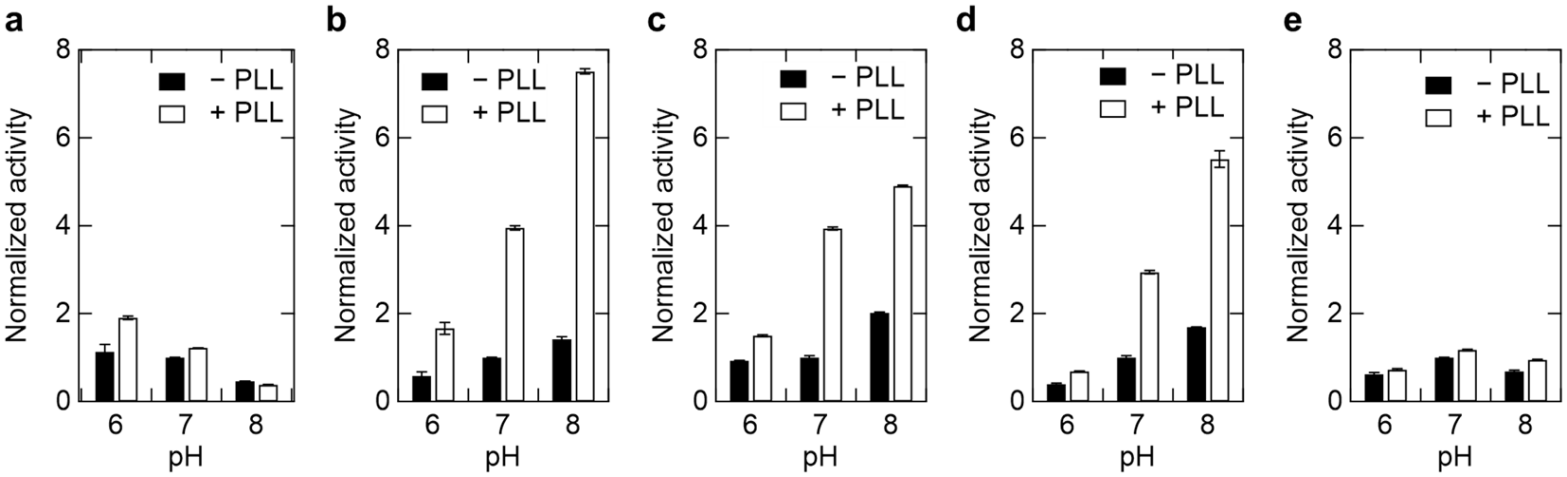
Enzymatic activity of oxidoreductases in the presence and absence of 1 mM Poly-l-lysine (PLL) at pH 6–8. (**a**) 100 nM POX, (**b**) 25 nM LOX, (**c**) 100 nM DLD, (**d**) 25 nM ALDH, and (**e**) 2.5 nM GDH. Enzyme activities in the absence of PLL at pH 7 were defined as 1. POX, Pyruvate oxidase; LOX, l-Lactate oxidase; DLD, d-Lactate dehydrogenase; ALDH, Aldehyde dehydrogenase; GDH, Glucose dehydrogenase.

**Figure 2:**
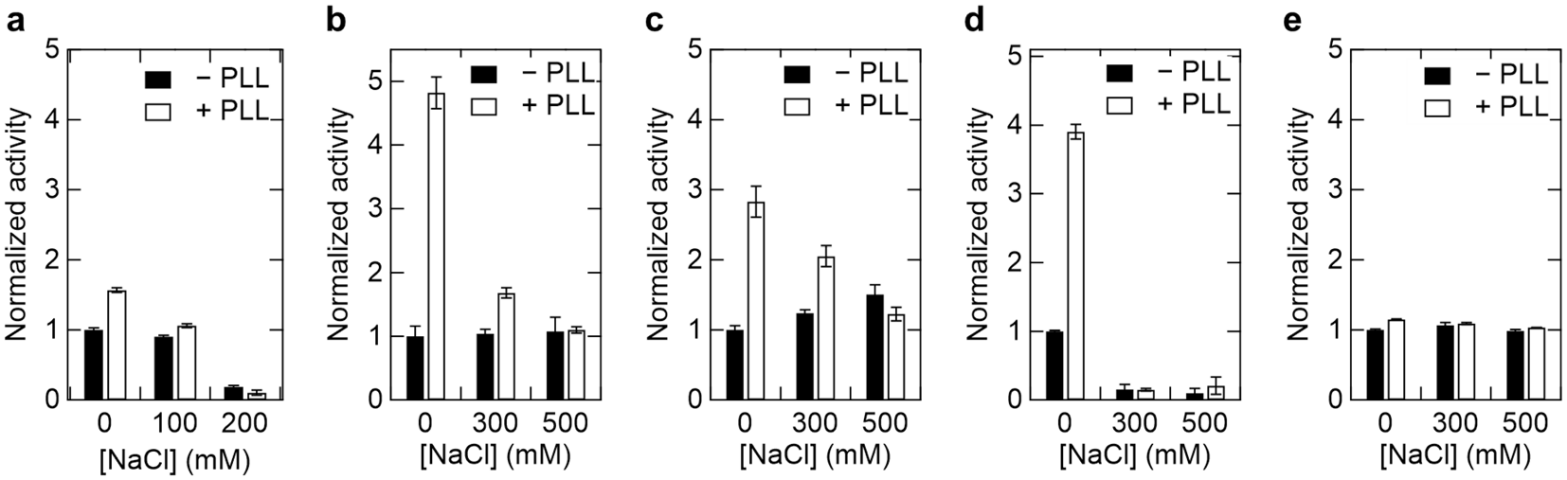
Effect of salt concentration on activity of oxidoreductases in the presence of PLL at pH 7. (**a**) 100 nM POX, (**b**) 25 nM LOX, (**c**) 100 nM DLD, (**d**) 25 nM ALDH, and (**e**) 2.5 nM GDH. PLL, Poly-l-lysine; POX, Pyruvate oxidase; LOX, l-Lactate oxidase; DLD, d-Lactate dehydrogenase; ALDH, Aldehyde dehydrogenase; GDH, Glucose dehydrogenase.

We used five oxidoreductases with different overall charges at physiological pH: l-lactate oxidase (LOX), acetaldehyde dehydrogenase (ALDH), d-lactate dehydrogenase (DLD), pyruvate oxidase (POX), and glucose dehydrogenase (GDH) (Table 1). At pH 7.0, ALDH and DLD are weakly negatively charged, LOX and POX are negatively charged, while GDH is positively charged. PLL was chosen as the additive polymer because it is positively charged at neutral pH (p*I* = 10) and expected to interact electrostatically with negatively charged enzymes. Figure 1 shows the pH-dependence of enzyme activities in the absence or presence of 1 mM PLL (monomer unit) at pH 6–8. The enzyme activity of POX increased moderately in the presence of PLL except for pH 8 (Fig. 1a), whereas in the presence of 1 mM PLL, the enzymatic activity of LOX was distinctly enhanced (Fig. 1b). The increase in activity of LOX following addition of PLL was approximately 4- and 7.5-fold at pH 7 and 8, respectively. In the presence of PLL, ALDH and DLD were also activated at pH 6–8 (Fig. 1c, d), while GDH was not activated (Fig. 1d). In addition, the effect of salt on PLL-induced enzyme activation was investigated since alterations in ionic concentration can change electrostatic interaction (Fig. 2). As the NaCl concentration is increased at pH 7, the enzyme activation effect in the presence of PLL attenuates above 300 mM and becomes equal to the activity similar to that in the absence of PLL above 500 mM (Fig. 2). POX showed a decreased activation effect and decreased enzymatic activity at lower NaCl concentrations (~ 200 mM) (Fig. 2a). In any case, all negatively charged enzymes have similar trends of NaCl concentration dependence, which correlated with pH dependence as shown in Fig. 1. These results suggest that the addition of PLL activates negatively charged oxidoreductases through electrostatic interactions between the enzyme and the PLL.

**Table 1:** Characteristics of enzymes used in this study.

Enzyme	Calculated p*I*^a^	Mass/kDa	Assembly state^b^	Coenzyme
Pyruvate oxidase (POX)	5.32	67.2	–	Flavin adenine dinucleotide (FAD)
L-Lactate oxidase (LOX)	5.66	40.6	Tetramer	Flavin mononucleotide (FMN)
D-Lactate dehydrogenase (DLD)	6.89	65.0	Tetramer	FAD
Aldehyde dehydrogenase (ALDH)	6.94	41.0	Monomer and dimer	Pyrroloquinoline quinone (PQQ)
Glucose dehydrogenase (GDH)	8.93	53.7	Tetramer	PQQ

^a^The p*I* of each enzyme was estimated from the amino acid sequence using ProtParam (https://web.expasy.org/protparam/).

^b^The assembly state of each enzyme was determined via size exclusion chromatography (Fig. S4), except for POX, which was difficult to measure due to nonspecific interaction to the column.

### Kinetic analysis of oxidoreductases in the absence or presence of PLL

**Figure 3:**
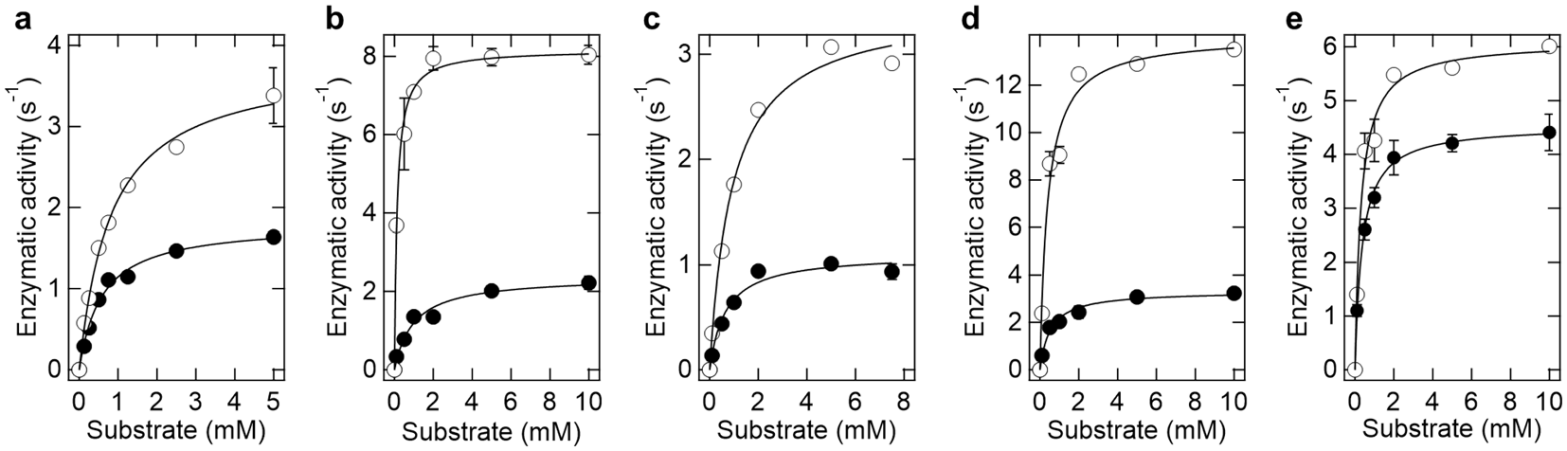
Enzyme kinetics of (**a**) 100 nM POX, (**b**) 25 nM LOX, (**c**) 100 nM DLD, (**d**) 25 nM ALDH, and (**e**) 2.5 nM GDH in the absence (filled circle) and presence of 1 mM PLL (open circle). PLL, Poly-l-lysine; POX, Pyruvate oxidase; LOX, l-Lactate oxidase; DLD, d-Lactate dehydrogenase; ALDH, Aldehyde dehydrogenase; GDH, Glucose dehydrogenase. Enzyme activity measurements were carried out at pH 7 and 25 ℃ and started by the addition of the respective substrate (l-lactic acid for LOX, acetaldehyde for ALDH, d-lactate for DLD, glucose for GDH, and pyruvic acid for POX).

To examine how the enzyme activity is modulated by PLL, the kinetic parameters were determined in 20 mM MES-Tris buffer (pH 7) at 25 °C (Table 2, Fig. 3). The *k*_cat_ values, indicating the catalytic turnover number, were higher in the presence of PLL than in its absence for all enzymes. Especially, LOX, ALDH, and DLD had several-fold higher activity. In turn, the *K*_M_ values, which indicates the affinity of the enzyme for its substrate, were dependent on the individual enzyme. The *K*_M_ of LOX and ALDH in the presence of PLL was approximately one seventh of that in the absence of PLL. The *K*_M_ value of DLD and POX in the presence of PLL was approximately 1.4-fold higher than that in the absence of PLL. The *K*_M_ values of GDH in the presence and absence of PLL were almost the same. Thus, PLL-induced increase in catalytic turnover was common to all enzymes, whereas the increase in substrate-enzyme affinity was enzyme dependent.

**Table 2:** Kinetic parameters of various oxidoreductases in presence and absence of PLL.

Enzyme	POX		LOX		DLD		ALDH		GDH	
PLL	0 mM	1 mM	0 mM	1 mM	0 mM	1 mM	0 mM	1 mM	0 mM	1 mM
*K*_M_ (mM)	0.58 ± 0.06	0.82 ± 0.07	0.98 ± 0.23	0.14 ± 0.02	0.66 ± 0.15	0.93 ± 0.13	0.53 ± 0.09	0.40 ± 0.08	0.36 ± 0.03	0.31 ± 0.05
*k*_cat_ (s^-1^)	1.81 ± 0.06	3.81 ± 0.11	2.38 ± 0.16	8.17 ± 0.16	1.1 ± 0.03	3.47 ± 0.06	3.31 ± 0.13	14.1 ± 0.06	4.54 ± 0.07	6.10 ± 0.19
*k*_cat_/*K*_M_	3.12	4.65	2.43	58.4	1.67	3.72	6.25	35.25	12.61	19.68

PLL, Poly-l-lysine; POX, Pyruvate oxidase; LOX, l-Lactate oxidase; DLD, d-Lactate dehydrogenase; ALDH, Aldehyde dehydrogenase; GDH, Glucose dehydrogenase.

### Formation of enzyme assemblies with PLL

**Figure 4:**
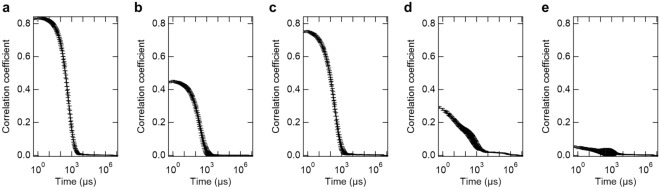
Autocorrelation functions from DLS measurement of sample solution containing PLL and enzymes (**a**) 100 nM POX, (**b**) 25 nM LOX, (**c**) 100 nM DLD, (**d**) 25 nM ALDH, and (**e**) 2.5 nM GDH. The hydrodynamic diameter of POX, LOX and DLD were calculated as 359.4 ± 26.6, 165.6 ± 7.5, and 264.2 ± 2.1 nm, respectively, by fitting. ALDH and GDH could not be fitted well due to high polydispersity. DLS, dynamic light scattering; PLL, Poly-l-lysine; POX, Pyruvate oxidase; LOX, l-Lactate oxidase; DLD, d-Lactate dehydrogenase; ALDH, Aldehyde dehydrogenase; GDH, Glucose dehydrogenase.

To investigate the relationship between the activation and formation of enzyme assemblies, we measured the sample solutions under the same conditions as in Fig. 1 via dynamic light scattering (DLS) (Fig. 4). The hydrodynamic radii of all enzymes in the absence of PLL could not be measured because of low scattering intensity. In the presence of PLL, autocorrelation functions could be detected (Fig. 4). For POX, LOX, and DLD, particles of 359.4 ± 26.6, 165.6 ± 7.5, and 264.2 ± 2.1 nm, respectively, were detected, which were considered to be enzyme assemblies. ALDH has a high polydispersity, and although the particle size was not uniquely determined, it is thought to form enzyme assemblies of various sizes. For GDH, the value of the correlation coefficient is considerably small; thus, it forms an even more unstable assembly. Furthermore, DLS measurements were not possible in the presence of NaCl above 300 mM due to polydispersity or low scattering intensity. This result correlates with the gradual attenuation of the activation effect with increasing NaCl concentration (Fig. 2). Thus, DLS results also indicate that the formation of enzyme assemblies with a size of hundreds of nanometers plays an important role in enzyme activation.

We investigated the correlation between the state of enzyme assemblies and activation rates in addition to assembly size obtained by DLS. This is because enzymes often become inactivated in an irreversible aggregated state but remain activated in the highly reversible droplet state^17^. Thus, the enzyme concentration was increased up to 5 μM, and the effects of pH and salt on the formation of enzyme assemblies were investigated using microscopy (Figs. S1 and S2). LOX and POX formed liquid droplet-like assemblies, whereas DLD formed aggregate-like assemblies between pH 6–8 (Fig. S1). The precipitation rate of enzyme in the presence of PLL showed that approximately 90–80% of POX, LOX, and DLD in solution were used in enzyme assembly (Fig. S3). LOX, DLD, and POX assemblies almost completely dissolved in 200 mM NaCl at pH 7 (Fig. S2); thus, these assemblies were stabilized by electrostatic interactions. Microscopically observable ALDH assemblies, containing approximately 20% of total ALDH, were much smaller, fewer in number, and reversible to NaCl (Figs. S1–S4). The formation of GDH assemblies in the presence of PLL could not be confirmed by microscopy (Fig. S1), and almost 100% of GDH remained a tetramer and did not change in solution (Figs. S3 and S4). The enzymatically active assemblies that formed were reversibly dissolved by the addition of a small amount of NaCl (Fig. S2). In contrast, POX-PLL assemblies at pH 8, which are likely enzymatically inactive assemblies, required 800 mM NaCl to dissolve completely (Fig. S6). In addition, these results suggest that the driving force for enzymatically active assembly formation is modest electrostatic interactions between the enzyme and PLL, rather than the shape of the enzyme assembly, which is important for enzyme activation.

### Secondary structure of enzyme in the enzyme assembly

**Figure 5:**
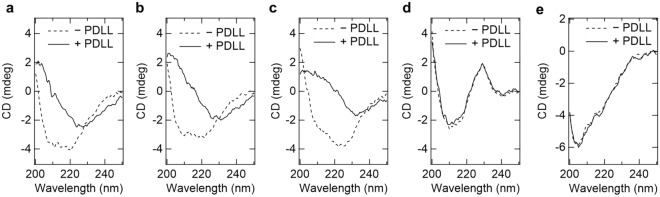
CD spectra of oxidoreductases in the presence of PDLL. Each sample solution contained 1 μM oxidoreductase (ALDH at 5 μM for sufficient absorption), 0.2 mM PDLL, and 20 mM Tris–HCl (pH 7). (**a**) POX, (**b**) LOX, (**c**) DLD, (**d**) ALDH, (**e**) GDH. PDLL, Poly-d-l-lysine; POX, Pyruvate oxidase; LOX, l-Lactate oxidase; DLD, d-Lactate dehydrogenase; ALDH, Aldehyde dehydrogenase.

We used far-UV circular dichroism (CD) spectroscopy to detect enzyme structural changes in the enzyme assemblies (Fig. 5). Poly-(d, l)-lysine (PDLL) was used in this experiment because it is not chiral and therefore eliminates the need to consider polymer-derived CD signals^14^. The far-UV spectra of POX, LOX, and DLD showed typical α-helix structures with negative ellipticity around 222 and 208 nm, respectively. In the presence of PDLL, the far-UV CD spectra of these enzymes changed to show negative ellipticity only around 230 nm. However, the positive ellipticity around 200 nm indicates that the conformation of the enzymes is not unfolded in the presence of PDLL. Thus, it is thought that the native conformation of the enzymes undergoes structural changes during enzyme assembly formation. In contrast, ALDH and GDH showed almost identical far-UV CD spectra in the presence or absence of PDLL.

## Discussion and conclusions

This study shows that the hundreds of nanometers of enzymatic assemblies formed by electrostatic interactions are linked to oxidoreductase activation, extending our previous work that examined only LOX in detail^14^. We investigated five types of oxidoreductases with different structures and surface charges to understand their characteristics that are likely to be activated in the enzyme assemblies. The overall charge of the proteins, which enables the electrostatic interaction with the PLL, is most important for activation. Thus, differences in activation rate among enzymes cannot be explained solely by the differences in the surface charges of the enzyme. The results of changes in pH (Fig. 1) and NaCl concentration (Fig. 2) indicate that the enzymatic activity is influenced by interaction with PLL.

Why does oxidoreductase activation occur when an enzyme assembly is formed? Kinetic parameters explain the effect of enzyme assemblies by an increase in the *k*_cat_ values for all tested enzymes (Fig. 3 and Table 2). A previous study suggested that changes in secondary structure may be associated with changes in *k*_cat_ or *K*_M_ of LOX^14^. Specifically, the perturbations in secondary structure associated with the formation of enzyme assemblies may reflect changes in the association state or the mobility of the dynamic loop site. POX and DLD showed a similar decrease in secondary structure as in LOX (Fig. 5). Similar to LOX, POX and DLD exist as tetramers^18,19^, and POX has a dynamic loop structure covering the active site^18^. Meanwhile, ALDH, which did not undergo significant secondary structure changes, was also activated. This may be a new mode of enzyme activation in enzyme assembly without conformational changes, possibly due to other factors such as cofactor differences (Table 1). Alternatively, given the high degree of polydispersity in the DLS results (Fig. 4), the conformation of the activated ALDH may be partial or highly labile, making it difficult to observe in CD. Even GDH, which did not undergo conformational changes and did not form enzyme assemblies, had a slight increase in *k*_cat_ (Table 2). The result of increased *k*_cat_ for all oxidoreductases suggested that the enzyme assembly or the PLL itself influenced each cofactor that mediates redox reactions. In contrast to the effect on *k*_cat_, the effect of enzyme assembly on *K*_M_ was enzyme dependent. It is considered that the frequency of non-specific adsorption of molecules increases as contaminants increase; thus, it is not surprising that the* K*_M_ of some enzymes slightly increases in the presence of PLL. To understand the effects of enzyme assembly on decreasing *K*_M_, physical factors such as changes in the collision frequency between substrates and enzymes should be considered. In other words, *K*_M_ is thought to decrease the most when the enzyme assembly enriches the substrate and excludes the product. Chemical factors such as changes in substrate-enzyme interactions also need to be investigated. Changes in the dielectric constant can occur in densely packed assemblies of proteins^20,21^, which could lead to changes in the electrostatic interactions between the substrate and the active site of the enzyme. The underlying mechanisms of this phenomenon remain to be elucidated, such as the influence of physicochemical parameters within the enzyme assembly on the enzymatic reaction mechanism, for instance, macromolecular crowding^22,23^, pH^24,25^ or relative permittivity^20,21^. In addition, since the enzyme used in this study forms multimers (Table 1), changes in multimers during the formation of enzyme assemblies may be important changes in *k*_cat_ or *K*_M_. Finally, there appears to be no direct correlation between the ease of assembly formation and the activation rate of enzyme. In other words, the fact that POX easily forms enzyme assemblies does not mean that the activation rate is high, but the opposite is true for ALDH. The reason for this may be related to the finding of a previous study showing that the smaller the size of the enzyme assembly, the higher the activation effect^14^. The claim of this study is that some enzymes share the feature of being activated in enzymatic assemblies compared to dispersed states. In any case, the fact that all the oxidoreductases tested were activated by forming enzyme assemblies with PLL compared to the dispersed state may be advantageous in terms of enzymatic application.

Although it is a well-observed phenomenon that enzymes and other macromolecules form assemblies in living cells, technological development based on this phenomenon has not progressed actively. Therefore, our research is important because it expands the knowledge on enzyme activation, especially oxidoreductases, via the addition of polymers, for which only limited reports are available using hydrolytic enzymes^26,27^. Since all the oxidoreductases tested in this study are expected to be applied to biofuel cells^28,29^ or biosensors^30,31^, their further improvement as a technology has a high industrial value. Furthermore, whether the results of this study can be applied to enzymes other than oxidoreductases may be an interesting topic for future research.

In summary, we demonstrated that oxidoreductases exhibit more enhanced activities in enzymatic assemblies than in dispersed states. Since this mimics reversible enzymatic assembly in cells, further pursuit of this phenomenon may lead to a better understanding of efficient enzymatic reactions in the cell. In addition, since it is a simple and easy method of just adding a polymer, it can be explored as a technique for activating enzymes in vitro.

## Material and methods

### Materials

Poly-l-lysine (PLL) hydrobromide (molecular weight (MW): 4000–15,000 Da), poly-(d, l)-Lysine (PDLL) hydrobromide (MW:25,000–40,000 Da), and 2,6-dichloroindophenol sodium salt hydrate (DCIP) were obtained from Sigma-Aldrich Co. (MO, USA). NaCl was obtained from Kanto Chemical Co., Inc. (Tokyo, Japan). Tris(hydroxymethyl)aminomethane, acetaldehyde (Tris), and glucose were obtained from Nacalai Tesque (Kyoto, Japan), and 2-morpholinoethanesulfonic acid monohydrate (MES) was obtained from Dojindo Laboratories (Kumamoto, Japan). l-lactic acid, d-lactic acid, and pyruvic acid were obtained from Tokyo Chemical Industry Co., Ltd. (Tokyo, Japan).

### Enzyme preparation

l-lactate oxidase (LOX) derived from *Enterococcus faecium* and acetaldehyde dehydrogenase (ALDH) from *Sphingomonas wittichii* were prepared as previously described^29,32^. LOX and ALDH were precipitated using 60% (saturation) ammonium sulfate. The obtained precipitates were suspended in a solution of 50 mM Tris (pH 7.5) and 3 M ammonium sulfate and stored as an ammonium sulfate suspension (20 mg/mL) at 4 °C until use. d-lactate dehydrogenase (DLD) from *Gluconobacter oxydans* was prepared as follows. The gene encoding DLD was amplified by polymerase chain reaction (PCR) from the genomic DNA of *G. oxydans* (JCM7642) using the primers 5′-gaaggagatatacatatgtcagcgtcactaaaaggcag-3′ and 5′- gagtgcggccgcaagcttagtggtggtggtggtggtggctgccgacgctctcggcaacccag-3′ and cloned into pET-22b vector. C-terminal histidine-tagged DLD was overexpressed in *Escherichia coli* BL21(DE3) and purified from cell extracts using TALON Metal Affinity Resin (Takara Bio, Shiga, Japan). DLD was then precipitated with 60% ammonium sulfate, and the pellet was suspended in 50 mM potassium phosphate (pH 7) with 3 M ammonium sulfate and stored as an ammonium sulfate suspension at 4 °C until use. Glucose dehydrogenase (GDH) from *Acinetobacter calcoaceticus* was prepared as follows. The expression vector encoding *A. calcoaceticus* GDH with six histidines at the C-terminus (pET22b-acGDH) was a gift from Aisin Cosmos R&D Co., Ltd (Aichi, Japan). The C-terminal histidine-tagged GDH was overexpressed in *E. coli* BL21(DE3) in LB medium containing 5 µM pyrroloquinoline quinone. The expressed GDH was purified from cell extracts using TALON Metal Affinity Resin (Takara Bio) and then precipitated with 60% ammonium sulfate. The GDH pellet was suspended in 20 mM Tris (pH 7.5), 1 mM calcium chloride, and 3 M ammonium sulfate, and stored as an ammonium sulfate suspension at 4 °C until use. Pyruvate oxidase (POX) from *Lactobacillus plantarum* was prepared as follows. The gene encoding POX (NCBI Reference Sequence: WP_003646223.1) with six histidines via a linker sequence (GSS) at the C-terminus was synthesized (Eurofins genomics, Kentucky, USA) and cloned into pET-22b (Novagen, Darmstadt, Germany). POX was overexpressed in *E. coli* BL21(DE3) and purified from the cell extracts using the TALON Metal Affinity Resin (Takara Bio). Then, POX was precipitated by ammonium sulfate at 60% saturation. The POX pellet was suspended in 50 mM Tris pH 7.5 and 3 M ammonium sulfate and stored as ammonium sulfate suspension at 4 °C until use. For each experiment, the enzyme stock solution was prepared by centrifuging a 50 µL suspension at 5000 × *g* for 5 min at 4 °C, removing the supernatant, and adding the buffer.

### Enzyme assays

The catalytic activities of the enzymes were measured.: Each enzyme solution containing the particular enzyme (25 nM for LOX and ALDH, 100 nM for DLD, 2.5 nM for GDH, and 100 nM POX), and 0–1 mM PLL (monomer concentration) in 20 mM buffer (MES and Tris–HCl) was prepared and incubated for 5 min at 25 °C. Protein concentration for each enzyme was determined by molar absorption coefficient (30,370 cm^−1^ M^−1^ for LOX, 42,448 cm^−1^ M^−1^ for ALDH, 61,810 cm^−1^ M^−1^ for DLD, 71,740 cm^−1^ M^−1^ for GDH, and 68,632 cm^−1^ M^−1^ for POX). A 160-μL aliquot of the enzyme solution was mixed with 40 μL aliquots of each substrate solution containing 0–10 mM substrates (l-lactic acid for LOX, acetaldehyde for ALDH, d-lactate for DLD, glucose for GDH, and pyruvic acid for POX), and 0.1 mM DCIP which has a molar extinction coefficient of 20,700 cm^−1^ M^−1^ at 600 nm^33^. The initial rate (*v*_0_) of activity was determined from the slope of the initial decrease in absorbance at 600 nm using an Ultrospec 2100 pro spectrophotometer (Biochrom WPA, UK) at 25 °C. The normalized enzymatic activity was defined as the ratio of *v*_0_ in the presence of PLL to that in the absence of PLL. All results are presented as the mean values of three independent experiments.

### DLS

DLS experiments were performed using a Zetasizer Nano ZS light scattering photometer (Malvern Instruments, Worcestershire, UK) equipped with a 4 mW-He–Ne ion laser (λ = 633 nm) at a detection angle of 173°. To determine the sizes of the enzyme complexes with polyelectrolytes, DLS of solutions containing 25–100 nM enzymes, 0–1 mM PLL, 20 mM MES-Tris buffer was measured with a 1 cm path-length disposable cuvette at 25 °C. The viscosities of the solutions were approximated using water (η = 0.87 cP). All results are presented as the mean values of three independent experiments.

### Optical microscopy

All images were recorded using a ZOE fluorescent cell imager microscope (Bio-Rad Laboratories, Inc., CA, USA). Microscopic images of the enzymes and PLL mixtures were obtained as follows: sample solutions were prepared by mixing 5 μM of each enzyme and 0–1 mM PLL solution in 20 mM MES-Tris buffer (pH 6–8) to a total volume of 150 µL. Sample solutions (150 μL) were plated on ultra-low attachment 96-well plates (Corning, NY, USA). Images were captured 1 h after mixing the solutions.

### CD

CD experiments were performed in a 1 cm path-length quartz cuvette using a spectropolarimeter (J-1100; JASCO Co., Ltd). Enzyme solution containing 1 μM LOX and 20 mM Tris–HCl buffer (pH 7.0) was incubated with 0.2 mM PDLL at 25 °C for 10 min before measurement. The CD spectra of the samples were corrected by subtracting the corresponding spectra of buffers.

### Precipitation rate of enzyme in the presence of PLL

Aliquots of 100 μL of solutions containing 5 µM enzyme and 1 mM PLL in 20 mM Tris–HCl buffer (pH 7.0) were centrifuged at 18,000 × g for 20 min at 25 ℃. The concentrations of enzyme in the supernatant were determined from absorbance at 280 nm using NanoPhotometer NP80 (Implen, Inc., CA, USA). Precipitation rates were calculated as Precipitation rate (%) = [1−C_n_/C_0_] × 100(%), where C_0_ is the enzyme concentration in the supernatant without PLL and C_n_ is the enzyme concentration in the supernatant with PLL.

### Gel filtration chromatography

Gel filtration chromatography was performed using the ÄKTA go™ system and Superdex 200 10/300 GL column (Cytiva, MA, USA). Injection of protein solution into a column and subsequent elution were performed with isocratic conditions. All experiments were performed at 25 °C at a flow rate of 0.75 ml min^−1^ in a buffer containing 150 mM NaCl, 0.1 mM DTT, 1 mM EDTA, and 25 mM Tris–HCl (pH 7.5). HPLC–UV chromatograms were recorded at 280 nm. After centrifugation, 300 μL of sample solutions was injected, and sufficient ultraviolet (UV) absorption was obtained. Gel filtration standard (Bio-Rad Laboratories, Inc.) was used as molecular weight standards.

### Supplementary Information

Supplementary Figures.

## Data Availability

Data is available upon reasonable request from the corresponding author.
