# Supplementary material for: Activation of oxidoreductases by the formation of enzyme assembly
Source: Sci Rep. 2023 Sep 1;13:14381. doi: 10.1038/s41598-023-41789-9 (PMC10474089; doi:10.1038/s41598-023-41789-9)
Supplement: Supplementary file 1 — Supplementary Figures. [file 41598_2023_41789_MOESM1_ESM.docx]

Supporting Information

Activation of oxidoreductases by the formation of enzyme assembly

Tomoto Ura^1,2,3^, Nanako Sakakibara^2,3^, Yu Hirano^1,4^, Taro Tamada^1,4^, Yoichi Takakusagi ^,4^, Kentaro Shiraki^2^, Tsutomu Mikawa^3,*^

^1^Institute for Quantum Life Science, National Institutes for Quantum Science and Technology, 4-9-1 Anagawa, Inage-Ku, Chiba 263-8555, Japan.

^2^Faculty of Pure and Applied Sciences, University of Tsukuba, 1-1-1 Tennodai, Tsukuba, Ibaraki 305-8573, Japan.

^3^RIKEN Center for Biosystems Dynamics Research, 1-7-22 Suehiro-cho, Tsurumi-ku, Yokohama 230-0045, Japan

^4^ Department of Quantum Life Science, Graduate School of Science, Chiba University, Yayoi-cho, Inage-Ku, Chiba 263-8522, Japan.

* To whom correspondence should be addressed.

Correspondence may also be addressed to Tsutomu Mikawa. Tel: +81-45-633-8013; Fax: +81-45-503-9643; Email: mikawa@riken.jp

T. Ura and N. Sakakibara contributed equally to this manuscript.


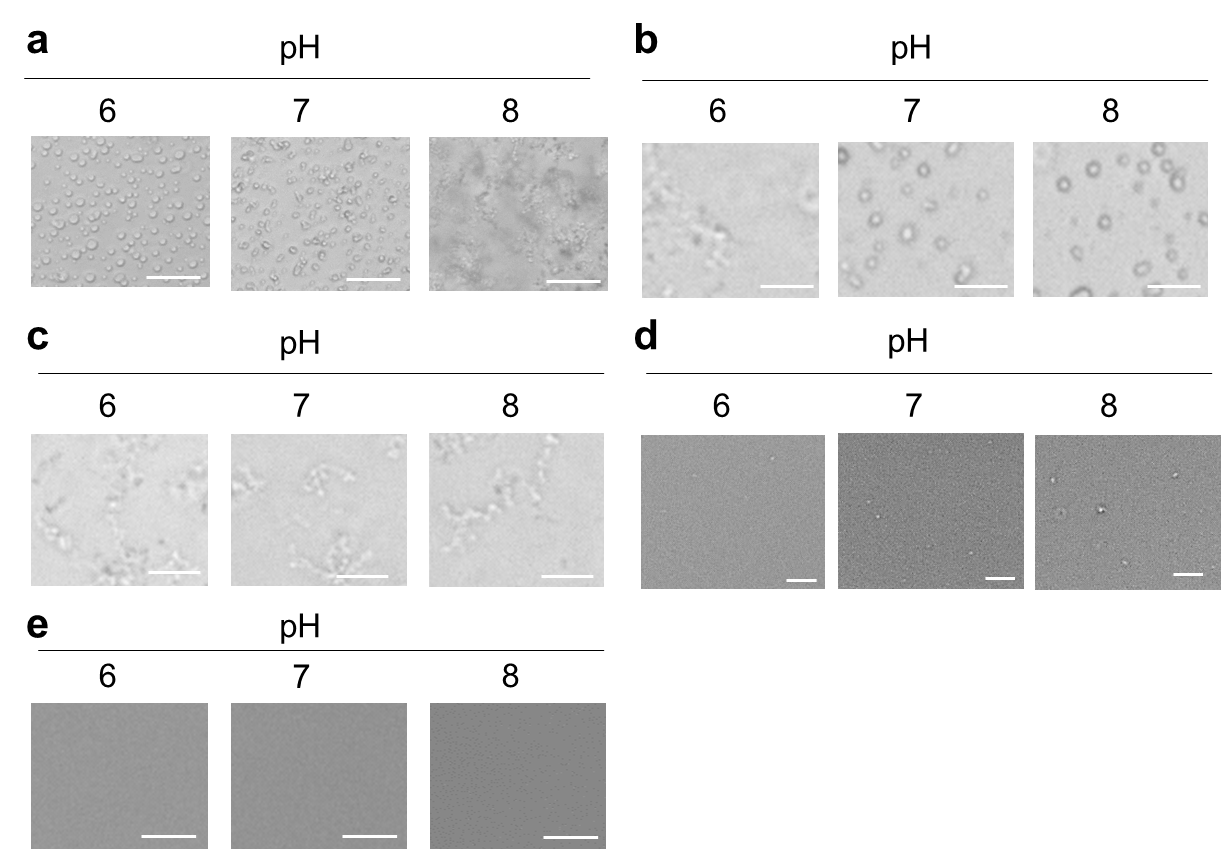


**Fig. S1**. Microscopic images of oxidoreductase-PLL assemblies at pH 6–8 (5 µM oxidoreductase, 1 mM PLL, and 20 mM MES-Tris buffer). (a) POX, (b) LOX, (c) DLD, (d) ALDH, (e) GDH. Scale bar = 10 µm. PLL, Poly-l-lysine; POX, Pyruvate oxidase; LOX, l-Lactate oxidase; DLD, d-Lactate dehydrogenase; ALDH, Aldehyde dehydrogenase; GDH, Glucose dehydrogenase.


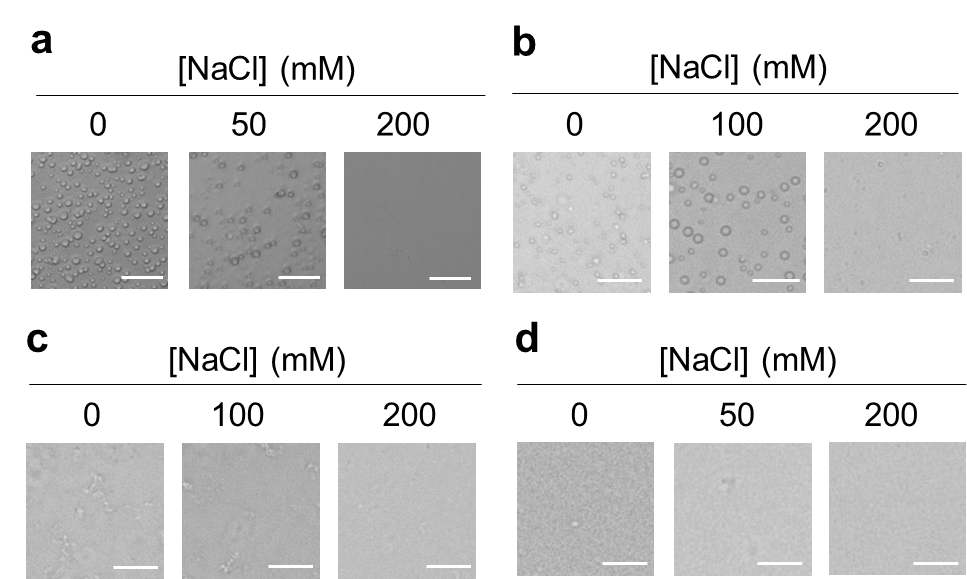


**Fig. S2**. Microscopic observation of oxidoreductase-PLL assemblies in the presence of NaCl (5 µM oxidoreductase, 1 mM PLL, and 20 mM MES-Tris buffer at pH 7). (a) POX, (b) LOX, (c) DLD, (d) ALDH. Scale bar = 10 µm. PLL, Poly-l-lysine; POX, Pyruvate oxidase; LOX, l-Lactate oxidase; DLD, d-Lactate dehydrogenase; ALDH, Aldehyde dehydrogenase; GDH, Glucose dehydrogenase.


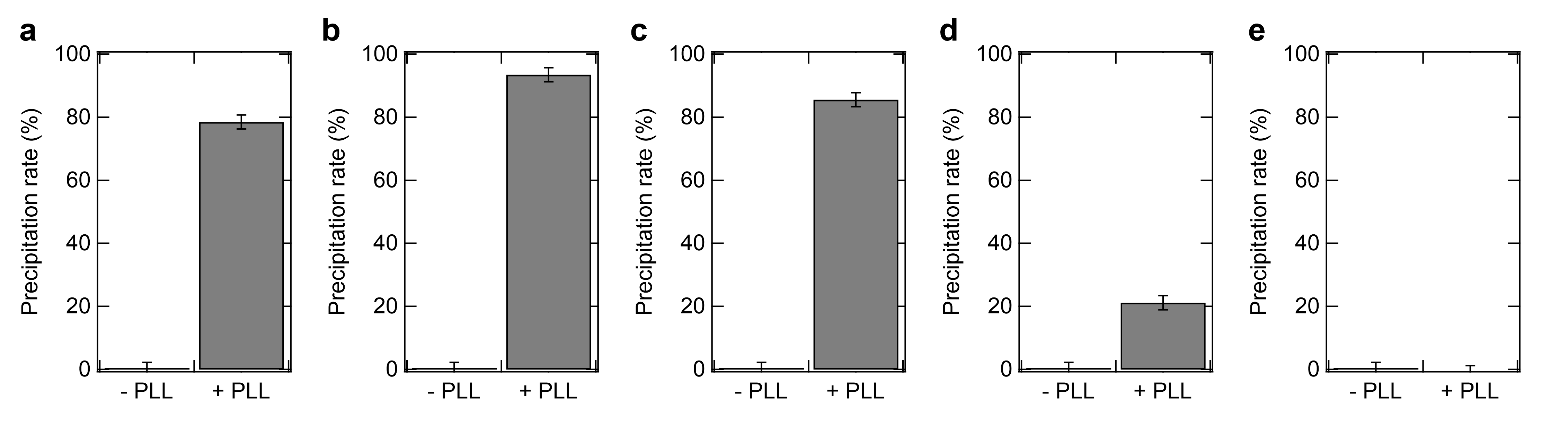


**Fig. S3** Precipitation rate (%) of each enzyme in the absence or presence of 1 mM PLL. (a) POX, (b) LOX, (c) DLD, (d) ALDH, and (e) GDH. All solutions were prepared with 5 µM enzyme, 0 or 1 mM PLL, and 20 mM Tris-HCl at pH 7.


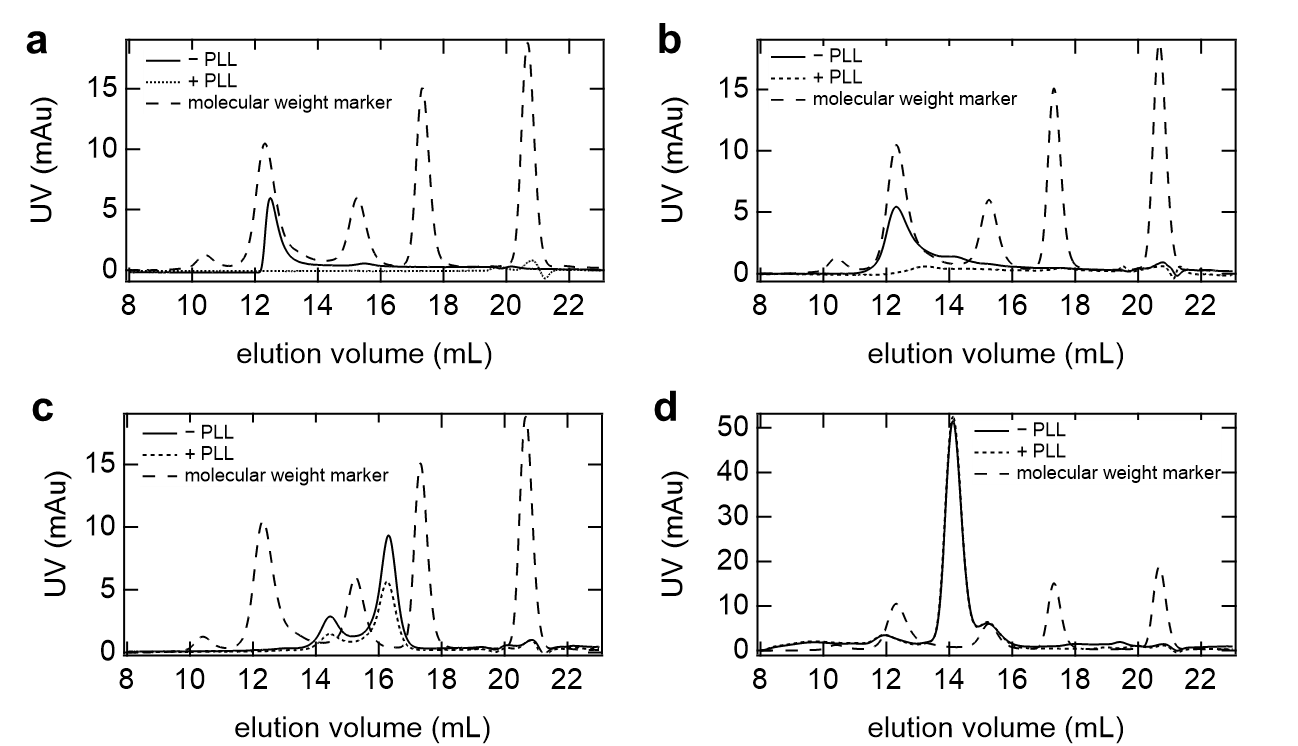


**Fig. S4** Gel filtration chromatography of the centrifugation supernatant of each enzyme solution in the absence or presence of 1 mM PLL. (a) LOX, (b) DLD, (c) ALDH, and (d) GDH. The molecular weight marker has five peaks with molecular weights of 670000, 158000, 44000, 17000, and 1350 from largest to smallest elution volumes.


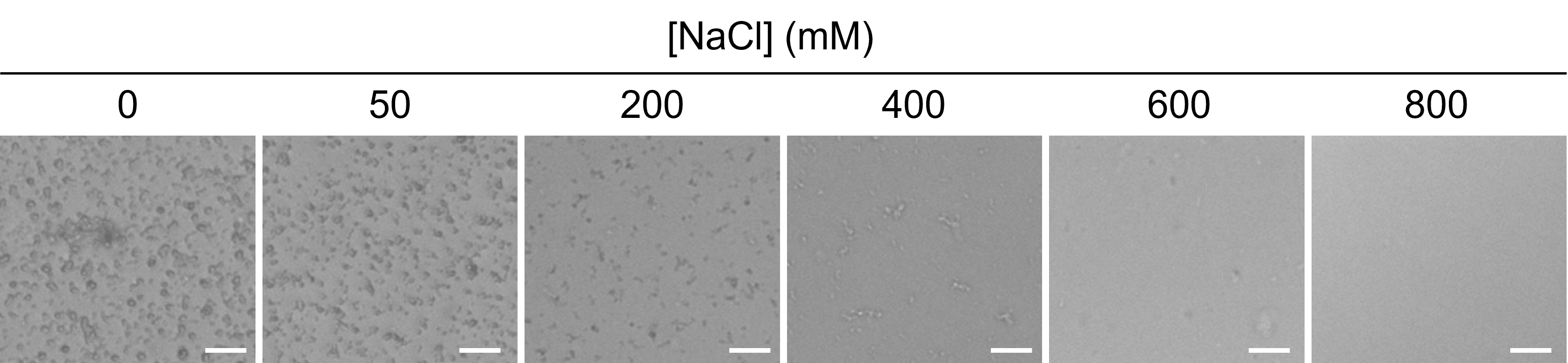


**Fig. S5** Microscopic observation of POX-PLL assemblies in the presence of 0-800 mM NaCl (5 µM POX, 1 mM PLL, and 20 mM MES-Tris buffer at pH 7). Scale bar = 20 µm
